# Tri-State Medical Association of Georgia, Alabama and Tennessee

**Published:** 1895-11

**Authors:** 


### TRI-STATE MEDICAL ASSOCIATION OF GEORGIA, ALABAMA AND TENNESSEE.

This Association, held in Chattanooga on the 9th, 10th and 11th insts., was a very interesting and profitable meeting. The following officers were elected for the ensuing year:

President, Dr. J. B. Murfree, of Murfreesboro, Tennessee; Vice Presidents, Dr. R. J. Trippe, Chattanooga, Tennessee, Dr. R. H. Hayes Union Springs, Alabama, Dr. R. R. Kime, Atlanta, Georgia; Secretary, Dr. Frank Trester Smith, Chattanooga, Tennessee; Treasurer, Dr. George R. West, Chattanooga, Tennessee.

The next meeting will be held in the City of Nashville, Tennessee, October, 1896.

---

THE American Association of Obstetricians and Gynæcologists held one of its most interesting and satisfactory meetings at Chicago, September 24th, 25th, and 26th, 1895. The attendance of members was large. Numerous papers were read relating to obstetrics, gynæcology and abdominal surgery, and the discussions, as usual in this Association, were spirited, forceful and instructive.

The President, Dr. J. Henry Carstens, of Detroit, administered the affairs of the Association with great discretion, facilitating the transaction of a large amount of business before it with thoroughness and dispatch.

The following named were elected officers for the ensuing year:

President, Dr. Joseph Price, of Philadelphia; Vice Presidents, Drs. Albert Hawes Cordier, of Kansas City, and George Sherman Peck, of Youngstown, Ohio; Secretary, Dr. William Warren Potter, of Buffalo; Treasurer, Dr. Xavier Oswald Werder of Pittsburg; Executive Council, Drs. Charles A. L. Reed of Cincinnati; James F. W. Ross, of Toronto; Albert Vander Veer, of Albany; Lewis S. McMurtry, of Louisville and J. Henry Carstens, of Detroit. Seventeen new fellows were also elected.

The ninth annual meeting was appointed to be held in Richmond, Virginia, Tuesday, Wednesday, and Thursday, September

15th, 16th, and 17th, 1896. Resolutions of thanks were passed as follows: First, to Dr. J. B. Murphy, chairman of the committee of arrangements, for the efficient manner in which he had provided for the comfort of the Fellows during the meeting and for the delightful yacht sail tendered the Fellows and guests of the Association; second, to the Chicago Gynæcological Society for many courtesies tendered; and third to Messrs. Breslin and Southgate, proprietors of the Auditorium hotel, for the free use of a splendid parlor, in which the meeting was held, and for courteous attention to the Fellows who were guests in the house.

---

THE Canton, Ohio, *News*, a daily newspaper, publishes the following truly remarkable account of what it fittingly describes as "A Rare Surgical Operation:"

Dr. J. S. Pyle, assisted by Drs. Harmount, House and Kelly, performed a very delicate operation at the Aultman Hospital yesterday morning. Mrs. William Lehnis, of 216 East Tuscarawas street, has been suffering for the past six months with a very severe attack of trifacial neuralgia. The left side of her face was so badly affected that if she started to talk it would cause her intense pain. It was decided to remove the muscles which caused the pain, as an only means of affording relief. A "V" shaped piece of scalp was laid back, extending from the base of the ear to the eye, and up to about two inches over the eye and back to the ear. The skull was then drilled through and pulled back, leaving the brain exposed. The brain was lifted, and this exposed the trifacial muscles. These were cut off and removed, the brain let down in position, and the bones of the skull pushed back. The scalp was then sewed up. The patient is feeling well this morning and the neuralgia is entirely gone. This is only the sixth time the operation has been performed in the United States, and then has only taken place in New York and Philadelphia.

